# Predictors of Self-rated Health Status Among Texas Residents

**Published:** 2005-09-15

**Authors:** Lorraine J Phillips, Renee L Hammock, Jimmy M Blanton

**Affiliations:** The University of Texas at Austin School of Nursing; The University of Texas at Austin School of Nursing, Austin, Tex; Behavioral Risk Factor Surveillance System Coordinator, Epidemiologist, Texas Department of State Health Services, Austin, Tex

## Abstract

**Introduction:**

The purpose of this study was to investigate the predictors of self-rated health status for Texas adults using the current 2003 Behavioral Risk Factor Surveillance System data. Self-rated health is generally accepted as a valid measure of health status in population studies, and understanding its correlates may help public health professionals prioritize health-promotion and disease-prevention interventions.

**Methods:**

The two research questions addressed by this study involved the predictors of self-rated health: 1) "Do demographic characteristics, health care coverage, leisure-time physical activity, and body mass index predict self-rated health status for Texas residents aged 18 to 64 years?" and 2) "Does choice of interview language (English vs Spanish) predict self-rated health status for Texas residents of Hispanic ethnicity aged 18 to 64 years?" Key analysis variables were identified, and descriptive statistics were used to describe the major variables and determine whether the number of respondents for each variable was sufficient for analysis. Multivariate regression analysis was used to assess the variables.

**Results:**

Multiple logistic regression analysis (controlling for diabetes and arthritis) of the self-rated health predictors indicated that older age, lack of health care coverage, lack of a college education, being Hispanic, having a lower income, obesity, and not exercising explained 19.4% of the variance of fair and poor self-rated health. The interview language (English or Spanish), age, sex, education, income, obesity, health insurance coverage, and physical activity (controlling for chronic illness) explained 22.8% of the variance in fair and poor self-rated health for Hispanic respondents.

**Conclusion:**

The results of this study suggest that a college education, a lower body mass index, non-Hispanic ethnicity, and participation in physical activity are associated with good, very good, or excellent self-rated health status. The finding that the interview language significantly predicted fair and poor self-rated health substantiates previous research and emphasizes the importance of culturally sensitive approaches to health care services.

## Introduction

The first goal of the U.S. Department of Health and Human Services' (DHHS's) *Healthy People 2010* is to help individuals in the United States improve their quality of life and life expectancy (1). With numerous other federal, state, and local agencies, DHHS monitors the health of individuals, communities, and the nation. When a particular health issue is identified, objectives that focus on strategies to reduce the severity of or eliminate the problem are developed, and many of these objectives are included in *Healthy People 2010*. *Healthy People 2010* also includes a model of health determinants that includes the following components: individual biology and behavior, individual social and physical environments, policies and interventions, and access to quality health care. All of these factors may interact with and affect the health of an individual or a society.

Improving the health of people living in the United States requires an initial assessment of their health status. Various instruments exist to measure perceived health. One such instrument is simply a question that asks people to rate their health as poor, fair, good, very good, or excellent. The Centers for Disease Control and Prevention (CDC) uses this self-reported global assessment of health-related quality of life in the annual Behavioral Risk Factor Surveillance System (BRFSS).

In population studies, self-rated health is generally accepted by researchers as a valid measure of health status. Because it is able to predict risk of death, self-rated health information measures not only psychological well-being but also overall health (2,3). Understanding the correlates of self-rated health may help health care professionals tailor health-promotion and disease-prevention interventions to the needs of specific populations. People who rate themselves as being in poor health tend to lack health care insurance (4-6), be women, be older, be black (7), and report lower psychological well-being (8). Alternatively, people who report that they are in good to excellent health tend to report higher vitality, a more positive mood, less vulnerability to illness (9), more frequent regular exercise (10,11), more education, and a higher income (7,12).

Previous studies have found that the relationship between a good or an excellent health rating and regular physical activity is stronger in men than women (8). In contrast, sex differences were not found between men and women in the same age group whose risk of death increased when they reported a lower level of physical fitness (13). Okosun et al (2) found that the association between obesity and less than excellent self-rated health was more pronounced in men than women, although a significant trend of fewer self-reports of excellent health with increases in obesity was found in both sexes and all racial/ethnic groups. Because obesity is associated with an increased risk of developing a chronic disease or condition, such as type 2 diabetes, high blood pressure, coronary heart disease, a high blood cholesterol level, osteoarthritis, or gallbladder disease (14), the lower self-reported health status ratings by obese individuals support the claim that self-rated health measures can reflect overall health.

A meta-analysis by Idler and Benyamini (15) showed that in 23 of 27 studies, self-ratings of health (independent of known health risk factors) reliably predicted survival, or life span, in the populations surveyed. The parsimonious global self-rating of health provides an invaluable and a unique assessment of health status. When respondents answer the question, "How in general would you rate your health?" the answer includes perceptions of their physical, mental, and social constitution. Whether self-rated health reveals unknown conditions, such as an undiagnosed disease, or is the most inclusive summary of all other influences on health (e.g., financial and personal resources, health behaviors, familial risk factors) is less relevant than its power to predict death (15). Because the correlation between self-rated health and mortality is well established, Idler and Benyamini also propose that future research on self-rated health status should focus on measures of morbidity, particularly those that increase mortality, such as new cases of heart disease, cancer, stroke, or diabetes.

The purpose of this study was to investigate the predictors of self-rated health status for Texas adults using 2003 BRFSS data. In 2003, Texas ranked fifth in the United States for the percentage of people who rated their health as fair or poor in the BRFSS; West Virginia ranked first, Mississippi second, Kentucky third, and Alabama fourth (16). In addition, Texas was second only to West Virginia in the percentage of people who reported having less than a high school education, and Texas residents reported less leisure-time physical activity than residents of 42 other states and the District of Columbia. Income levels tended to be lower in Texas, with only seven other states reporting higher percentages of households earning less than $25,000. Finally, in 2003, Texas had the highest percentage of uninsured people in the nation, with 26.6% reporting a lack of health care coverage (17).

The Hispanic/Latino population in Texas comprises 32% of the total population (18), increasing the chance that the typical categorical responses of all Texans on a self-rated health status scale will have cross-cultural differences. In other words, the adjectives associated with normal health may differ between Hispanics and non-Hispanics. An analysis of the Hispanic Health and Nutrition Examination Survey (HHANES) revealed that the language used by the interviewer has a significant effect on self-ratings of health (19). Angel and Guarnaccia (19) reported that respondents who were interviewed in Spanish were much less likely to report excellent health (15%) or good health (48%) than people who were interviewed in English. Of respondents interviewed in Spanish, almost half of the people who rated their health as fair or poor were rated by a physician as having very good or excellent health, suggesting that level of acculturation (measured by language of interview) significantly affects self-ratings of health. Given the prevalence of potential predictors of fair or poor self-rated health in Texas, identification of these factors may help guide the direction of future research and health-promotion interventions.

Based on findings in the available research, we developed the following questions for this study:

1. While controlling for chronic illness, do demographic characteristics, health care coverage, leisure-time physical activity, and body mass index (BMI) predict self-rated health status for Texas residents aged 18 to 64 years? (Arthritis and diabetes were chosen to represent the chronic disease state.)
2. While controlling for chronic illness, does choice of interview language (English vs Spanish) predict self-rated health status for Texas residents of Hispanic ethnicity aged 18 to 64 years?

## Methods

Our study was an analysis of the 2003 BRFSS data. The BRFSS — a state-based, ongoing telephone survey of persons aged 18 years and older — links behavior risk factors to chronic illness in the adult population. State health departments conduct the survey in conjunction with the CDC. Participants are selected using a random-digit–dialing method to gather a representative sample of noninstitutionalized adults. The data were weighted and poststratified to adjust for demographic differences between the sample and known Texas demographics. The sample of interest for this study was adults aged 18 to 64 years who were residing in Texas (N = 4091).

The BRFSS has three sections:

1. *Core questions,* which are asked in every state in the same order, using the same precise instructions. In 2003, the BRFSS core had 20 question modules including topics such as respondent demographics, health status, access to care, and exercise frequency.
2. *Optional question modules,* which are modules that are supported by the CDC and are optional for each state. The optional modules are typically used to gather in-depth information about a specific subject such as asthma, diabetes, or tobacco use.
3. *Additional questions*, which are developed and added by each state. In 2003, the state-added questions used by Texas were related to vitamin use, physical activity, and weight loss.

The data for our analysis involved only questions from the core module. The dependent variable in the study — self-rated health — was measured by the question, "Would you say that in general your health is excellent, very good, good, fair, or poor?"

Key analysis variables identified for the study included the following: 1) age (18 to 44 years and 45 to 64 years); 2) health care coverage (yes or no); 3) education (less than high school, high school graduate or some college, and college graduate); 4) sex (male or female); 5) race/ethnicity (white, black, Hispanic, or other); 6) household income (<$25,000, $25,000 to $74,999, or ≥$75,000); 7) BMI, calculated from weight and height (BMI = kg/m^2^) — not obese (BMI <30) or obese (BMI ≥30); 8) whether physical activity or exercise other than that involved in a regular job had been performed in the past month (yes or no); 9) interview language for people of Hispanic ethnicity (English or Spanish); and 10) self-rated health status (excellent, very good, good, fair, or poor).

To accommodate the complex sampling design of the BRFSS, data analysis was performed using SPSS, version 12.0 (SPSS Inc, Chicago, Ill) in conjunction with SUDAAN (Research Triangle Institute, Research Triangle Park, NC). Descriptive statistics were used to describe the major variables and determine whether the number of respondents for each variable was sufficient for analysis. To address the first research question ("Do demographic characteristics, health care coverage, leisure-time physical activity, and BMI predict self-rated health status for Texas residents aged 18 to 64 years?"), a multivariate logistic regression analysis was used to assess self-rated health while controlling for chronic illness. As predictor variables, the analysis included the variables that significantly correlated with the dependent variable (self-rated health). Household income, education, exercise, and BMI were found to correlate strongly with self-rated health, as were race/ethnicity, health care coverage, and age. Because marital status did not have a statistically significant correlation with self-rated health, the variable was not included in the final model. We controlled for the confounding influence of chronic illness on the explanatory power of the logistic model by including arthritis and diabetes that had been diagnosed by a physician. In our study, the dependent variable was dichotomized into two categories: 1) fair/poor health and 2) good/very good/excellent health. The second research question ("Does choice of interview language [English vs Spanish] predict self-rated health status for Texas residents of Hispanic ethnicity aged 18 to 64 years?") was also addressed by multivariate logistic regression analysis (while controlling for chronic disease), with descriptive statistics included for reference. Statistical significance for both analyses was set at *P* <.001.

## Results

Table 1 presents the distribution of the sample among the categories of the dependent variable, self-rated health. Overall, older respondents, women, respondents from households with an income of less than $25,000 per year, obese individuals, and respondents who participated in no exercise other than that required to perform their job rated their health as poor. Respondents who rated their health as excellent were younger, had health care coverage, had a college degree, were white, had a household income of greater than $75,000 per year, were of a normal weight, and reported participating in physical activity or exercise other than that required in their regular job. Most of the respondents classified themselves as white, had a high school education, and had health care coverage. Although the majority of respondents reported an annual household income greater than $25,000, a separate analysis of income by ethnicity revealed that 24.8% of Hispanic respondents reported an annual income of less than $15,000 in 2003.

Table 2 is a summary of the multiple logistic regression analysis results for the predictors of fair/poor self-rated health. The analysis shows that (when controlling for diabetes and arthritis) older age, lack of health care coverage, having less than a college education, having a Hispanic ethnicity, having a lower income, being obese, and not exercising explained 19.4% of the variance of fair/poor self-rated health (*R* ^2^ = 0.1942). Sex and a race/ethnicity designation of black or "other" were not significantly associated with fair/poor health.

For an additional test of the impact of culture and interview language on self-rated health, a multiple logistic regression was used to analyze respondents of Hispanic ethnicity (Table 3). The following independent variables were included: choice of interview language, age, sex, education, income, BMI, health insurance coverage, and physical activity. The final model controlled for chronic illness, did not include the sex variable, and explained 22.8% of the variance in fair/poor self-rated health (*R* ^2^ = 0.2281). The participants who chose to be interviewed in Spanish were significantly more likely to rate their health as fair/poor than were participants who chose English.

## Discussion

The results of this study suggest that higher education, a lower BMI, non-Hispanic ethnicity, and participation in physical activity are consistently associated with good, very good, or excellent health status. Because education, BMI, and physical activity are modifiable, these findings underscore the importance of including physical activity and nutrition education in public health programs. For instance, in the United States in 2003, medical costs attributable to obesity were estimated as being $75 billion (20); the Texas estimated costs were $5.34 billion (20). Being overweight significantly increases the risk of developing a chronic illness (21). Women with a BMI greater than 35 were 17 times more likely to develop diabetes than were women with a BMI of less than 25, and men with a BMI greater than 35 were 23 times more likely to develop diabetes than were men with a BMI of less than 25 (21). Effective weight control and reduction programs not only may save billions of U.S. health care dollars but also may reduce the incidence of chronic disease associated with obesity.

Brown et al (22) assessed the association between levels of physical activity and health-related quality of life and found that the relative odds of having 14 or more unhealthy days (physically or mentally unhealthy) were significantly lower for people who met recommended levels of physical activity than for physically inactive adults across all age, racial/ethnic, and sex groups. Collectively, poor diet and physical inactivity were second only to tobacco use as the leading cause of death in the United States in 2000 (23). A lifestyle with a poor diet and physical inactivity not only increases risk of death but also results in years of lost life, diminished productivity, high rates of disability, and a decreased quality of life (23). The results of our study concur with Mokdad et al's assessment that fair/poor self-rated health was related to a lack of exercise and obesity.

Higher education and income levels have been linked to better health in individuals (12). For example, in an 8-year longitudinal study of a Chicago neighborhood, Browning et al found that when income and education were included in the health status model, health improved across time in relation to reported education and income (12); Browning et al did not report a temporal association between unemployment and poor health-related quality of life. However, low income (i.e., less than $15,000 per year per household) was associated with worse health-related quality of life for men and women aged 45 to 64 years (24). Employment status and activity limitation accounted for the most variability in number of unhealthy days.

The results, which indicated that the interview language significantly predicted fair/poor self-rated health, substantiate previous research studies. Angel and Guarnaccia (19) found that level of acculturation, which was measured by the interview language chosen by the participant, was independently correlated with the respondent's subjective assessment of health. One possible explanation was that the adjectives used to describe normal health for Mexican Americans and Puerto Ricans differed from those used by people who were not of Hispanic origin. In addition, lower acculturation was associated with a tendency to express distress somatically, which was evidenced by higher scores on standard depressive affect scales in the study (19). The authors highlight the importance of social and cultural influences on bodily perceptions, which must be considered when comparing subjective health levels among various social and cultural groups (19).

We found that the most powerful predictors of self-rated health are the predictors that are potentially modifiable. Of respondents that were not obese, 86.7% reported being in good to excellent health; in contrast, 74.6% of participants in the obese category reported being in good to excellent health. Of the modifiable predictors of self-rated health, weight may be the most realistically changeable factor. Exercise is extremely important for controlling BMI. Of the respondents who reported being in excellent health, the highest percentage exercised regularly. Of those reporting poor health, the highest percentage did not exercise regularly. Many health care providers are highly respected by their patients — individuals who may be at risk for developing lifestyle-related chronic diseases. Health care providers should seize the opportunity to address their patients' weight issues and sedentary lifestyles. They should stress the need for exercise and weight control to increase quality of life.

In addition, the importance of an education — at the very least, a high school education — should be emphasized to adolescents and their parents as vitally important to their future health. Culturally sensitive approaches to health care services and delivery also must be considered when caring for individuals of various ethnic backgrounds, because as our findings suggest, health perceptions are influenced not only by medical factors but also by sociocultural factors.

## Figures and Tables

**Table 1:** Self-rated Health Status by Demographic and Health-related Characteristics, Texas, 2003^a^

**Characteristics**	**No. Respondents**	**Self-rated Health Status (%)**					

		**Excellent**	**Very Good**	**Good**	**Fair**	**Poor**	**Total**

**Age, y**							

All ages (18-64)	4091	22.2	31.0	30.3	12.8	3.7	100
18-44	2395	24.4	31.9	30.6	11.3	1.9	100
45-64	1696	18.3	29.2	29.8	15.6	7.0	100

**Health care coverage**							

Yes	3070	24.0	34.3	29.4	9.1	3.2	100
No	1021	17.6	22.3	32.6	22.5	5.0	100

**Education**							

Less than high school	485	10.0	13.2	35.1	34.3	7.4	100
High school graduate or some college	2192	20.4	30.8	33.4	11.5	3.9	100
College graduate	1414	31.4	39.9	22.4	4.7	1.6	100

**Sex**							

Male	1692	23.4	30.6	30.5	12.7	2.9	100
Female	2399	20.9	31.4	30.1	13.0	4.6	100

**Race/ethnicity**							

White	2557	25.9	35.5	26.7	8.3	3.7	100
Black	383	18.8	26.4	37.1	12.8	5.0	100
Hispanic	1012	15.6	23.8	35.3	21.6	3.7	100
Other	139	26.0	28.6	29.9	14.4	1.0	100

**Household income**							

<$25,000	1236	13.8	20.2	34.9	23.7	7.4	100
$25,000-$74,999	1867	21.9	34.5	31.8	9.3	2.5	100
≥75,000	988	34.2	38.8	21.1	4.9	1.0	100

**Body mass index (BMI)**							

Not obese (BMI <30)	3005	26.1	33.0	27.6	10.4	3.0	100
Obese (BMI ≥30)	1086	11.4	25.2	38.0	19.7	5.8	100

**Exercise other than required at work**							

Yes	3128	25.2	34.2	29.2	9.3	2.1	100
No	963	13.0	20.9	33.7	23.9	8.6	100

**Doctor-diagnosed diabetes**							

Yes	282	4.1	17.3	27.7	31.9	19.1	100
No	3809	23.5	32.0	30.5	11.5	2.6	100

**Doctor-diagnosed arthritis**							

Yes	917	12.2	26.8	30.6	19.3	11.2	100
No	3174	24.8	32.0	30.2	11.2	1.8	100

a Table includes only records with complete information for all variables in the logistic regression model. Records with missing values for any of the model variables are not analyzed.

**Table 2:** Fair/Poor Self-rated Health and Adjusted Odds Ratios (AOR) for Selected Characteristics, Texas, 2003

**Characteristics**	**Overall Percentage**	**Percentage With Fair/Poor Health (95% CI)^a^**	**AOR** **(95% CI)^a^,^b^**	***P* Value**

**Age, y**				

All ages (18-64)	100.0	16.5 (15.2-17.9)		
18-44	64.4	13.1 (11.6-14.9)	Reference	
45-64	35.6	22.6 (20.3-25.1)	1.74 (1.35-2.23)	<.001

**Health care coverage**				

Yes	72.0	12.2 (11.0-13.6)	Reference	
No	28.0	27.5 (24.3-31.0)	1.51 (1.15-1.98)	.003

**Education**				

Less than high school	14.8	41.7 (36.6-47.0)	4.56 (3.08-6.75)	<.001
High school graduate or some college	54.6	15.4 (13.8-17.2)	1.47 (1.09-1.99)	.012
College graduate	30.6	6.3 (5.1-7.8)	Reference	

**Sex**				

Male	53.5	15.6 (13.7-17.7)	0.95 (0.75-1.19)	.633
Female	46.5	17.6 (15.9-19.4)	Reference	

**Race/ethnicity**				

White	57.9	11.9 (10.6-13.4)	Reference	
Black	8.9	17.8 (13.7-22.9)	1.00 (0.65-1.55)	.991
Hispanic	29.4	25.3 (22.2-28.7)	1.39 (1.05-1.83)	.023
Other	3.8	15.5 (9.5-24.2)	1.73 (0.93-3.22)	.086

**Household income**				

<$25,000	31.7	31.1 (28.0-34.3)	3.01 (2.03-4.48)	<.001
$25,000-$74,999	45.0	11.8 (10.1-13.6)	1.52 (1.07-2.17)	.021
≥$75,000	23.3	5.9 (4.5-7.7)	Reference	

**Body mass index (BMI)**				

Not obese (BMI <30)	73.8	13.4 (11.9-14.9)	Reference	
Obese (BMI ≥30)	26.2	25.5 (22.6-28.5)	1.56 (1.23-1.98)	<.001

**Exercise other than required at work**				

Yes	75.8	11.4 (10.2-12.8)	Reference	
No	24.2	32.5 (29.0-36.2)	2.18 (1.72-2.77)	<.001

**Doctor-diagnosed diabetes**				

Yes	6.7	51.0(44.2-57.7)	4.60 (3.26-6.50)	<.001
No	93.3	14.0 (12.8-15.4)	Reference	

**Doctor-diagnosed arthritis**				

Yes	20.1	30.5 (27.1-34.0)	3.00 (2.33-3.86)	<.001
No	79.9	13.0 (11.6-14.5)	Reference	

a CI indicates confidence interval.

b Adjusted for all other variables in Table 2. N = 4091 before weighting.

**Table 3:** Interview Language, Fair/Poor Self-rated Health, and Adjusted Odds Ratios (AOR) Among Hispanic Respondents, Texas, 2003

	**Overall Percentage**	**Percentage With Fair/Poor Health (95% CI)^a^**	**AOR ** **(95% CI)^a^,^b^**	***P* Value**
All ages (total Hispanic sample, aged 18-64 years)	100.0	27.4 (24.6-30.4)		
Hispanics interviewed in Spanish	46.5	38.7 (34.0-43.6)	2.96 (2.21-3.96)	<.001
Hispanics interviewed in English	53.5	17.6 (14.8-20.8)	Reference	

a CI indicates confidence interval.

b Adjusted for all other variables in Table 3. N = 1315 before weighting.
